# Larviciding intervention targeting malaria vectors also affects *Culex* mosquito distribution in the city of Yaoundé, Cameroon

**DOI:** 10.1016/j.crpvbd.2023.100136

**Published:** 2023-07-28

**Authors:** Abdou Talipouo, Patricia Doumbe-Belisse, Carmène S. Ngadjeu, Landre Djamouko-Djonkam, Elysée Nchoutpouen, Roland Bamou, Nadège Sonhafouo-Chiana, Audrey Paul Marie Mayi, Gisèle Aurélie Dadji Foko, Parfait Awono-Ambene, Sévilor Kekeunou, Charles S. Wondji, Christophe Antonio-Nkondjio

**Affiliations:** a Institut de Recherche de Yaoundé (IRY), Organisation de Coordination pour la lutte Contre les Endémies en Afrique Centrale (OCEAC), P.O. Box 288, Yaoundé, Cameroon; b Faculty of Sciences, University of Yaoundé I, P.O. Box 337, Yaoundé, Cameroon; c Faculty of Sciences, University of Dschang, Box 337, Dschang, Cameroon; d Centre for Research in Infectious Disease (CRID), Yaoundé, P.O. Box 13591, Cameroon; e Faculty of Health Sciences, University of Buea, Buea, Cameroon; f Laboratory of Zoology, Higher Teacher Training College, University of Yaoundé I, P.O. Box 47, Yaoundé, Cameroon; g Vector Biology Liverpool School of Tropical Medicine Pembroke Place, Liverpool, L3 5QA, UK

**Keywords:** Larviciding, *Culex*, *Bacillus thuringiensis*, *Bacillus sphaericus*, Yaoundé

## Abstract

Although *Culex* species are considered to be equally affected by control measures targeting malaria vectors, there is still not enough evidence of the impact of interventions such as larviciding on the distribution of these mosquito species. The present study assessed the impact of a larviciding trial targeting malaria vectors on *Culex* mosquito species in the city of Yaoundé, Cameroon. A cluster randomized trial comparing 13 treated clusters and 13 untreated clusters was implemented. Data were collected at baseline and during the larviciding intervention, from March 2017 to November 2020. The microbial larvicide VectoMax G was applied once every 2 weeks in the intervention areas. Adult mosquitoes were collected using CDC light traps in both intervention and non-intervention areas and compared between arms. Globally, larviciding intervention was associated with 69% reduction in aquatic habitats with *Culex* larvae and 36.65% reduction of adult *Culex* densities in houses. Adult *Culex* densities were reduced both indoors (35.26%) and outdoors (42.37%). No change in the composition of *Culex* species was recorded. The study suggests a high impact of larviciding on *Culex* mosquito species distribution. The impact of the intervention can be improved if typical *Culex* breeding habitats including pit latrines are targeted.

## Highlights

• Larviciding targeting malaria vectors also affects *Culex* mosquito densities.
• No change in the composition of *Culex* species was recorded.
• Larviciding could help control *Culex* mosquitoes nuisance.

## Graphical abstract

**Figure:**
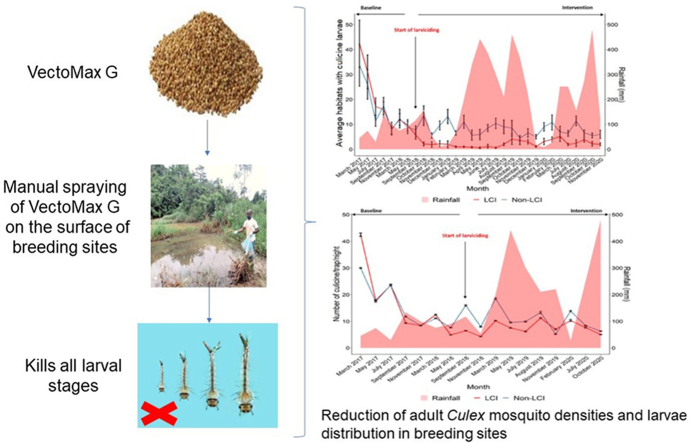

## 1 Introduction

The rapid unplanned urbanization in most African countries has important consequences for hygiene and public health (Knudsen and Slooff, 1992). Like most major sub-Saharan African cities, the city of Yaoundé is experiencing a rapid demographic growth mainly due to the massive migration of the population from rural to the urban areas. This demographic growth has as an immediate consequence the expansion of slums in the city centre, absence of adequate sanitation infrastructure, and exploitation of lowland areas for urban agriculture and house construction. This situation has led to the creation of suitable breeding conditions for mosquitoes, particularly for *Culex* species.

Although *Culex* mosquitoes are not vectors of human malaria, they appear as potential vectors of diseases such as arboviruses and filariasis (Rift Valley fever, Sindbis, Wesselsbron, o'nyong-nyong, and West Nile virus and Bancroftian filariasis) that affect more than 1 billion people globally (Amraoui et al., 2012; Diallo et al., 2014; Phumee et al., 2019; Mayi et al., 2020). Most of these pathogens are maintained in zoonotic cycles with humans being incidental hosts. Thus, due to their opportunistic behaviour, *Culex* species could act as bridge vectors increasing the risk of transmission of new pathogens to humans (Matowo et al., 2019).

Vector control could be recommended for fighting *Culex* species and diseases that they transmit. Vector control measures in Cameroon mainly rely on the use of long-lasting insecticidal nets (LLINs). This insecticide-based tool is highly threatened by the rapid expansion of insecticide resistance in vector populations. In addition to anopheline species (Etang et al., 2016; Antonio-Nkondjio et al., 2017; Mandeng et al., 2019), high insecticide resistance levels mediated by different mechanisms have been reported in *Culex* mosquitoes (Nchoutpouen et al., 2019; Talipouo et al., 2021), stressing the need to implement additional vector control interventions.

Larval control has been proven to be a promising tool for vector control or mosquito abatement programmes (WHO, 2013) and could be a good complement to existing interventions. Several studies across Africa have shown a high efficacy of larviciding for malaria vector control (Fillinger et al., 2009; Tusting, 2014; Dambach et al., 2019). In Cameroon, two pilot larval control trials against *Culex quinquefasciatus* have so far been conducted. Larviciding intervention in Maroua using a liquid formulation of *Bacillus sphaericus* strain 2362 consisting of two treatments per year of all water collections had a limited impact on *Cx. quinquefasciatus* biting densities (Barbazan et al., 1997). A pilot study in Yaoundé using the same product reported a 64% reduction rate of *Cx. quinquefasciatus* biting densities (Hougard et al., 1993). However, these findings should be taken with caution since the study was subjected to a certain number of bias (Hougard et al., 1993; Antonio-Nkondjio et al., 2019).

In the city of Yaoundé, *Culex* larvae are found in various types of breeding habitats including stagnant water collections, gutters, wells, tyre prints, footprints and pit latrines (Nchoutpouen et al., 2019). In the majority of these breeding habitats, *Culex* larvae can be found in sympatry with anopheline larvae (Antonio-Nkondjio et al., 2019; Nchoutpouen et al., 2019). Therefore, we hypothesized that larviciding targeting anopheline larvae could equally affect *Culex* mosquito species distribution. The present study comes in complement to a previous study which assessed the impact of larviciding on anopheline mosquito biting densities and malaria transmission in the city of Yaoundé, Cameroon (Antonio-Nkondjio et al., 2021). The goal of the present study is to assess the impact of the intervention on *Culex* mosquitoes.

## 2 Materials and methods

### 2.1 Study sites

The present study was carried out in Yaoundé (3°51′N, 11°29′E) the capital city of Cameroon, one of the largest cities of Cameroon with over 3 million inhabitants. Yaoundé features an equatorial climate with four seasons: two rainy seasons extending from March to June and from September to November and two dry seasons extending from July to August and December to February. Yaoundé is located 800 m above sea level with a landscape dominated by lowland and highland areas. Lowlands are exploited during the dry season for urban agriculture. The majority of the breeding habitats in Yaoundé (> 90%) are located in lowland areas. Study sites were located in lowlands, highly populated areas with poor drainage, high pollution and the presence of numerous standing water collections full of organic matter. The study was designed as a cluster randomized trial comprising 26 clusters of 2–4 km^2^ (Antonio-Nkondjio et al., 2021). Thirteen clusters were selected after randomization to be treated (larviciding intervention area) and the other thirteen served as control (larviciding non-intervention area).

### 2.2 Larvicidal intervention

**Fig. 1:**
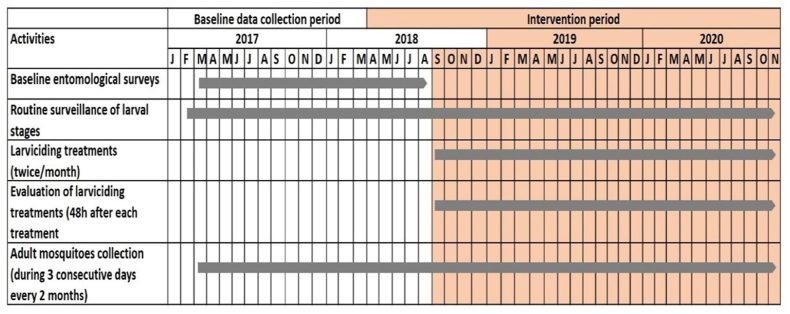
Comprehensive timeline of the programmatic steps taken in the present study.

In the 13 intervention area clusters, the larvicide used was VectoMax G (Valent Biosciences Corporation, USA); a granule formulation containing both *Bacillus thurengiensis israelensis* (*Bti*), strain AM65-52 (45 g/kg), and *B. sphaericus* (*Bsph*), strain ABTS-1743 (27 g/kg). All standing water collections and containers found with water in the larviciding intervention area were treated once every two weeks with VectoMax G granules at the dosage of 500–1500 mg/m^2^. All water collections were systematically treated even if mosquito larvae were not found in them. More details about the treatment strategy are available elsewhere (Antonio-Nkondjio et al., 2021). The study was conducted following the chronogram shown in Fig. 1.

### 2.3 Follow-up of *Culex* larval habitats

In the larviciding intervention area, water collections were checked 48 h after each treatment to find mosquito larvae in order to assess the effectiveness of treatments. Additional site inspections were done 7 or 8 days after each treatment to detect the creation of new breeding habitats. In the larviciding non-intervention area, breeding sites were also checked for the presence of mosquito larvae once every month. The presence of mosquito larvae was checked using the standard dipping method (Service, 1993). Up to 10 dips were performed, depending on the size of the water body. Three dips were performed for small breeding sites of less than 1 m^2^, and 5–10 dips in breeding sites of more than 1 m^2^ (Nchoutpouen et al., 2019). A given breeding site was considered as positive when at least one immature mosquito stage was found. All breeding sites were georeferenced using a Garmin eTrex® GPS and recorded in a GIS database for analysis. The effect of larviciding efficacy was assessed by the use of CDC-LT to determine the density of adult *Culex* mosquitoes. The secondary outcome was the proportion of breeding habitats containing *Culex* larvae.

### 2.4 Adult mosquito collection

Adult mosquitoes were collected once every 2 months from March 2017 to November 2020 using the Center for Diseases Control Light traps (CDC-LTs). For each collection period, 15 to 20 CDC-LTs were used per cluster each night. Collections were performed from 19:00 to 6:00 h in 10–15 randomly selected houses per cluster during 3 consecutive nights.

### 2.5 Mosquito identification

Following collection, *Culex* mosquitoes were separated from anophelines and sorted by species using morphological identification keys (Edwards, 1941; Jupp, 1996). Species belonging to *Cx. pipiens* complex were further processed by PCR to distinguish between *Cx. pipiens*, *Cx. quinquefasciatus* and *Cx. pallens* using the loci CQ11. For molecular identification, DNA extracted from whole mosquitoes according to the method described by Livak (1984) was used to run a multiplex PCR assay according to Smith and Fonseca (2004). PCR amplification reactions were carried out in a 15 μl volume reaction mix, containing 10 × PCR buffer, 250 μM of each dNTP, 1.7 mM MgCl_2_, 0.15 mM of bovine serum albumin, 1-unit Taq polymerase (Applied Biosystems, Waltham, USA), 2 μl of genomic DNA, and 11.6 nM of each primer. The primers used were: ACEpip (5′-GGA AAC AAC GAC GTA TGT ACT-3′); ACEpall (5′-ATG GTG GAG ACG CAT GAC G-3′); ACEquin (5′-CCT TCT TGA ATG GCT GTG GCA-3′); and B1246s (5′-TGG AGC CTC CTC TTC ACG G-3′). The PCR products were then separated by electrophoresis on 1.5% agarose gel with Midori green and visualized under ultraviolet light.

### 2.6 Data analysis

Data were recorded in Excel. Linear Mixed Models with random intercepts and Generalized Estimating Equations (GEE) were used to evaluate the effect of larviciding on breeding habitats with *Culex* larvae and adult *Culex* densities, adjusting for baseline data. In preliminary analysis, follow-up curves for the larviciding non-intervention and intervention groups were constructed to visualize differences in the responses between the two groups. Average trends and local polynomial regressions of proportion of breeding habitats with *Culex* larvae *versus* date were also constructed for the different groups to further visualize these differences. In linear mixed modelling, we first estimated a null model with random intercept and calculated the intraclass correlation coefficient associated with these respective proportions. GEE were further used to assess the impact of larviciding on *Culex* larvae presence in aquatic habitats with clustering by water body and zone included as random effects. GEE analyses were also used to assess the impact of larviciding on adult *Culex* densities by treating larviciding as a categorical independent factor in the model. Comparisons were adjusted for survey periods (months), years, baseline densities and clustering by traps and cluster. In all these cases, the identity link function with a Gaussian distribution was used, and we resorted to a model with independent correlation structures. Clusters were treated as the geographical location, year as the indicator of time, larval presence in aquatic habitats and *Culex* densities as means for each cluster over the full year or the duration of the intervention. Although in the present analysis clusters were used as the experimental units for the analysis which allowed the impact of larviciding to be estimated, some individual factors operating at the house level were also assessed. We control for individual level factors such as houses by treating individual houses as experimental units and preventing cluster larviciding covariance by restricting our analysis to the 466 houses used for mosquito collection surveyed during both the baseline and intervention period. A first order autoregressive relationship was applied for all repeated measurements. All analyses were performed with R 4.0.2 software using the R packages *nlme*, *lm4*, *ggplot2*, *plyr*, *lattice*, *car*, *effects*, *emmeans* and *data.table*. The percentage reduction of mosquito density and proportion of breeding habitats with *Culex* larvae following the larviciding intervention were estimated using the formula of Mulla et al. (1971). The Shannon index measuring species diversity was also calculated and compared between baseline and intervention periods.

## 3 Results

### 3.1 Distribution of *Culex* larval habitats before and during larviciding intervention

Larval collections were done at baseline and during the implementation of larviciding to capture the evolution of breeding habitats containing *Culex* larvae. Before the implementation of larviciding, there was no significant difference (*P* = 0.129) in the proportion of larval habitats containing *Culex* larvae between the larviciding intervention and non-intervention areas. During larviciding intervention, the proportion of larval habitats containing *Culex* larvae was significantly higher (*P* < 0.0001) in the larviciding non-intervention area compared to the larviciding intervention area. Overall, the proportion of larval habitats containing *Culex* larvae was reduced by up to 69% in the larviciding intervention area following the implementation of larviciding (Table 1).

**Table 1:** Distribution of breeding habitats with *Culex* spp. larvae at baseline and during the larviciding intervention.

	Baseline		Intervention		Percent reduction
	Larviciding non-intervention area	Larviciding intervention area	Larviciding non-intervention area	Larviciding intervention area	
Total no. of water bodies checked	8313	8633	25,729	137,120	
Total no. of water bodies with *Culex* larvae	1528	1773	2523	5538	69.24
Proportion (%) of positive breeding habitats (95% CI)	18.38 (17.47–19.33)	20.54 (19.59–21.52)	9.80 (9.42–10.19)	4.03 (3.93–4.14)	
*P*-value (Non-LCI *vs* LCI)	0.129		< 0.0001		

*Note:* Percent reduction = 100 – (Non-LCI at baseline/LCI at baseline × LCI during intervention/non-LCI during intervention) × 100.

*Abbreviations*: LCI, larviciding intervention area; Non-LCI, larviciding non-intervention area.

**Fig. 2:**
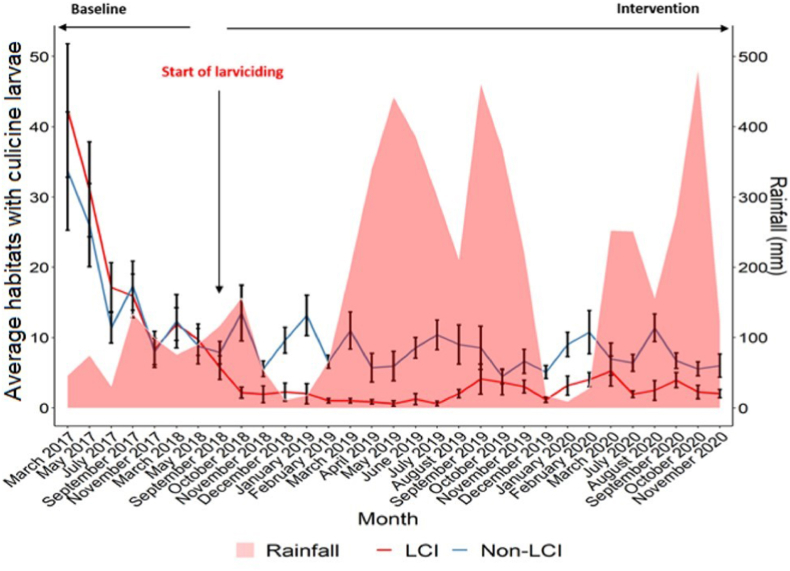
Distribution of breeding habitats with *Culex* larvae before and during larviciding intervention. Error bars represent 95% CI. *Abbreviations*: LCI, larviciding intervention area; Non-LCI, larviciding non-intervention area.

Further analysis indicated that from March 2017 to June 2018 before the implementation of larviciding, there was no significant reduction in mean number of breeding sites with *Culex* larvae between the larviciding intervention and non-intervention areas. During larviciding, a significant reduction of breeding habitats with *Culex* larvae was recorded in the larviciding intervention area compared to the larviciding non-intervention area regardless of the month of study. The monthly distribution of breeding habitats with *Culex* larvae was found to vary according to the rainfall pattern (Fig. 2).

### 3.2 Evolution of proportion of breeding habitats with *Culex* larvae

A mixed linear modelling approach was used to better assess the impact of the intervention. For the modelling analysis, 1131 measurements in both larviciding intervention and non-intervention areas were taken into consideration. Mixed logistic regression model analysis showed no significant differences in the proportion of breeding habitats with *Culex* larvae in both the larviciding intervention and non-intervention groups at baseline. However, a significant reduction in the proportion of breeding habitats with *Culex* larvae in the larviciding intervention group compared to the non-intervention was recorded during larviciding treatments (Table 2).

**Table 2:** Mixed effects logistic regression models of the effect of intervention on the proportion of breeding habitats with *Culex* larvae, controlling for date and season at baseline and during larviciding intervention.

Parameter	Baseline		Intervention	
	OR (95% CI)	*P*-value	OR (95% CI)	*P*-value
*Fixed effects*				
Intercept	0.056 (0.039–0.081)	<0.0001	0.1190 (0.092–0.154)	<0.0001
Date (month)	0.904 (0.893–0.915)	<0.0001	1.003 (0.997–1.008)	0.334
Group (Reference: Non-LCI)				
LCI	0.994 (0.590–1.675)	0.9810	0.118 (0.082–0.171)	<0.0001
Season (Reference: Dry)				
Rainy	No dry season data		0.700 (0.657–0.747)	<0.0001
Date: Group (Reference: Non-LCI)				
LCI	0.999 (0.982–1.017)	0.9140	1.018 (1.010–1.027)	0.00004
*Random effects*				
Cluster random effects variance	0.351		0.198	

*Abbreviations*: LCI, larviciding intervention area; Non-LCI, larviciding non-intervention area; CI, confidence interval; OR, odds ratio.

Further analysis of the results showed that the estimated marginal mean of the proportion of breeding habitats with *Culex* larvae, using GEE were not significantly different between the larviciding intervention and non-intervention areas at baseline. However, during larviciding intervention, GEE results showed a significant reduction in the proportion of breeding habitats with *Culex* larvae in the larviciding intervention area compared to the larviciding non-intervention area. The estimated mean proportion of breeding habitats with *Culex* larvae at the beginning, midway and at the end of the larviciding intervention were significantly lower in the larviciding intervention area than in the non-intervention area (Table 3).

**Table 3:** Estimated marginal means of the proportion of breeding sites with *Culex* larvae from the Generalized Estimating Equations (GEE) models, adjusted for baseline survey, period and group.

Date	Group	Baseline		Intervention	
		Estimated Mean (95% CI)	*P*-value	Estimated Mean (95% CI)	*P*-value
Beginning of study	Non-LCI	26.86 (22.00–31.71)	0.8791	9.88 (7.87–11.89)	<0.0001
	LCI	27.47 (21.20–33.75)		1.39 (0.89–1.88)	
Midway	Non-LCI	19.17 (16.15–22.19)	0.7113	10.11 (9.10–11.13)	<0.0001
	LCI	19.97 (17.03–22.91)		1.79 (1.54–2.04)	
End of study	Non-LCI	9.07 (4.76–13.37)	0.7350	10.42 (8.18–12.65)	<0.0001
	LCI	10.10 (5.95–14.25)		2.31 (1.82–2.80)	

*Abbreviations*: LCI, larviciding intervention area; Non-LCI, larviciding non-intervention area; CI, confidence interval.

### 3.3 *Culex* species distribution before and during the larviciding intervention

**Fig. 3:**
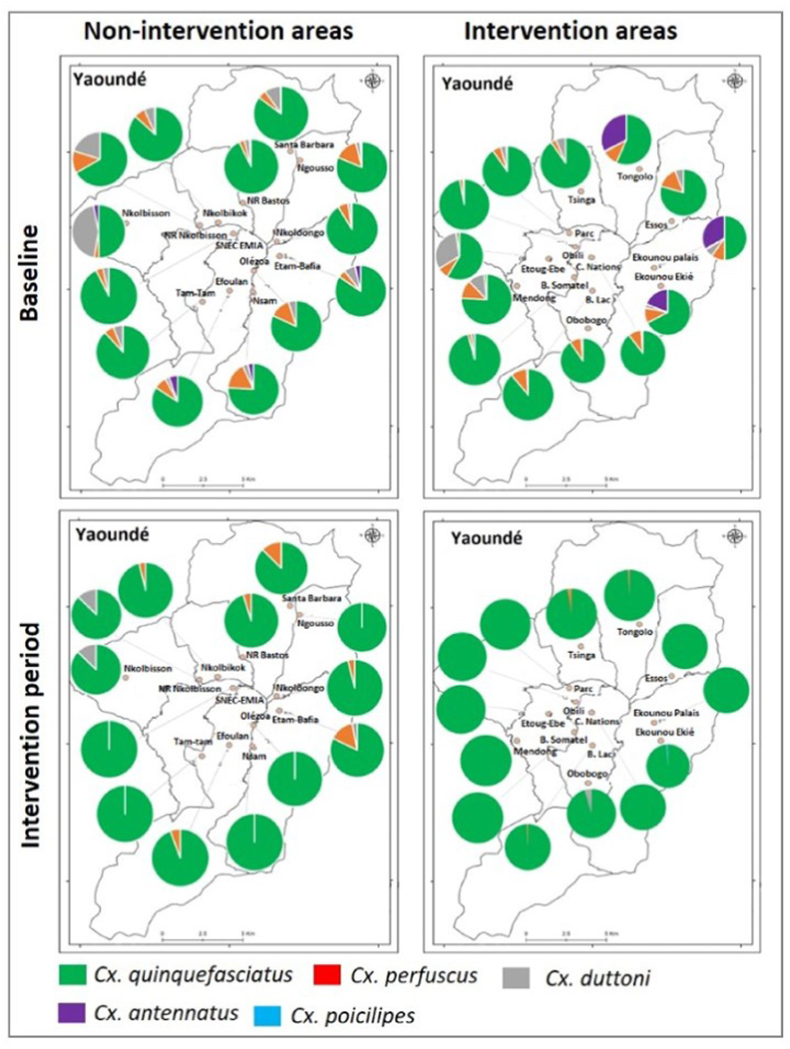
Composition and distribution of *Culex* species before and during the larviciding trial in Yaoundé, Cameroon.

A total of 291,679 *Culex* mosquitoes were collected during the study. Out of this number, 20,125 specimens were identified down to species level using morphological identification keys. A subsample of 261 specimens morphologically identified as belonging to the *Cx. pipiens* complex were further processed for molecular identification using PCR and confirmed as *Cx. quinquefasciatus*. *Culex* species collected included *Cx. quinquefasciatus*, *Cx. perfuscus*, *Cx. duttoni*, *Cx. antennatus* and *Cx. poicilipes* (Fig. 3). In all districts prospected, *Cx. quinquefasciatus* was the predominant species. Variation in the composition of *Culex* species before and during the larviciding intervention was recorded in both larviciding intervention and non-intervention areas. *Culex quinquefasciatus* was the only species recorded in most intervention areas after the launch of the larviciding intervention (Fig. 3). In larviciding intervention clusters, the Shannon diversity index was significantly higher (*P* < 0.001) at baseline (H’ = 0.85) compared to intervention period (H’ = 0.0542) suggesting a decrease in the diversity of *Culex* species with larviciding activities. In larviciding non-intervention areas, the Shannon index varied from H’ = 0.7668 during baseline studies to H’ = 0.1189 during the intervention period and the difference was not significant.

### 3.4 Evolution of adult mosquito densities

Globally, larviciding was associated with 36.65% reduction of adult *Culex* densities. The density of mosquitoes collected both indoors and outdoors were reduced (Table 4). The average density of *Culex* mosquitoes collected in the larviciding non-intervention area varied from 15.67 mosquitoes per trap per night at baseline to 10.89 mosquitoes per trap per night during the larviciding intervention. In the larviciding intervention area, the average density of *Culex* mosquitoes collected varied from 17.20 mosquitoes per trap per night at baseline to 7.58 mosquitoes per trap per night during the larviciding intervention (Table 4).

**Table 4:** Distribution of adult *Culex* densities at baseline and during the larviciding intervention.

Parameter	Baseline		Intervention		Percent reduction
	Larviciding non-intervention area	Larviciding intervention area	Larviciding non-intervention area	Larviciding intervention area	
**Total no. of *Culex* collected**	82,002	90,304	70,497	48,876	
**Total no. of traps**	5233	5250	6468	6447	
**Mean no. of *Culex/*trap/night (95% CI)**	15.67 (15.56–15.77)	17.20 (17.08–17.31)	10.89 (10.81–10.98)	7.58 (7.51–7.64)	36.65
**Mean no. indoors (95% CI)**	20.82 (20.66–20.98)	22.04 (21.89–22.20)	15.7 (15.57–15.83)	10.76 (10.66–10.90)	35.26
**Mean no. outdoors (95% CI)**	7.91 (7.79–8.03)	8.40 (8.27–8.54)	4.33 (4.25–4.41)	2.65 (2.59–2.71)	42.37

*Note*: Percent reduction = 100 – (Non LCI at baseline/LCI at baseline × LCI during intervention/non-LCI during intervention) × 100.

*Abbreviations*: LCI, larviciding intervention area; Non-LCI, larviciding non-intervention area; CI, confidence interval.

**Fig. 4:**
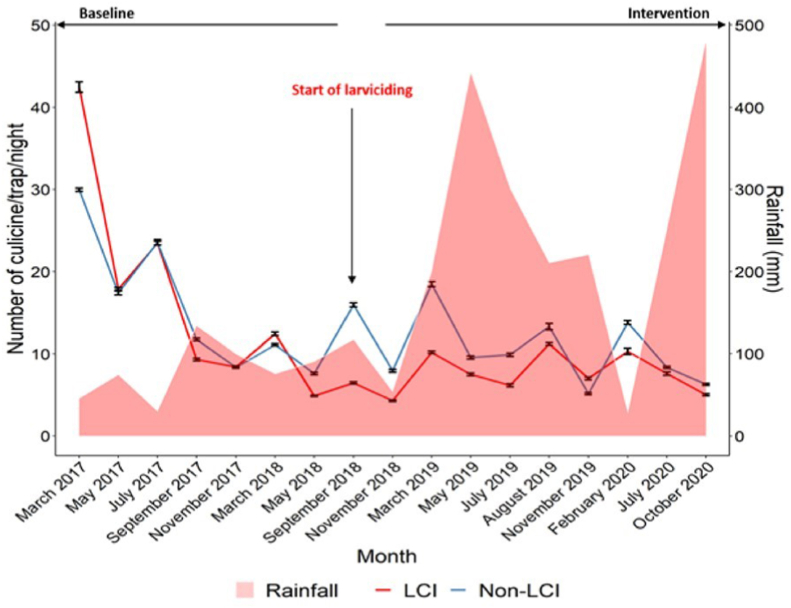
Variation of adult *Culex* mosquito densities before and during larviciding intervention. Error bars represent 95% CI. *Abbreviations*: LCI, larviciding intervention area; Non-LCI, larviciding non-intervention area.

In general, both larviciding intervention and non-intervention areas showed similar patterns of *Culex* mosquito density at baseline (Fig. 4). After launching the larviciding intervention, *Culex* mosquito densities were found to decrease drastically in the larviciding intervention area compared to the non-intervention area, supporting a significant impact of larviciding on these mosquitoes. The monthly distribution of adult *Culex* mosquito densities varied according to the rainfall pattern and treatment regime (Fig. 4).

A linear mixed model was applied to assess the effect of larviciding on adult *Culex* mosquito densities. Mosquito densities in the larviciding intervention area compared to the non-intervention area were similar at baseline. During larviciding intervention, a significant reduction was recorded with *Culex* densities significantly reduced in the larviciding intervention area compared to the non-larviciding area after controlling for period (Table 5).

**Table 5:** Mixed linear model of the effect of larviciding on the adult *Culex* mosquito densities at baseline and during larviciding intervention.

Parameters	Baseline		Intervention	
	Estimate (95% CI)	*P*-value	Estimate (95% CI)	*P*-value
***Fixed effects***				
**Intercept**	16.327 (6.481, 26.173)		10.867 (8.684, 13.051)	
**Group (Reference: Non-LCI)**				
**LCI**	1.931 (-2.705, 6.568)	0.334	-3.299 (-5.507, -1.091)	0.001
***Random effects***				
**Date (Month)**				
**Variance**	98.90		5.687	

LCI, larviciding intervention area; Non-LCI, larviciding non-intervention area; CI, confidence interval.

## 4 Discussion

The main objective of the study was to assess the impact of a larviciding trial targeting malaria vectors on *Culex* mosquito distribution in the city of Yaoundé. The larval control trial was associated with 36% reduction of adult *Culex* mosquito densities. Similar reductions have been reported previously in Burkina Faso and Tanzania (Geissbühler et al., 2009; Dambach et al., 2021). The low reduction rate compared to anophelines (Antonio-Nkondjio et al., 2021) could be associated to the complex ecology of *Culex* mosquitoes in urban settings. *Culex* mosquitoes are known to breed in polluted environment including septic tanks, pit latrines and polluted puddles (Nchoutpouen et al., 2019). The majority of breeding habitats such as pit latrines located in private properties were not targeted by larviciding activities. It is also possible that the level of pollution, presence of vegetation, solid containers or waste in drains, may have reduced the efficacy of larviciding treatments in these areas by providing numerous hiding places for *Culex* larvae.

In this study, mosquitoes were collected using the Center for Disease Control Light traps (CDC LT). This permitted to minimize the inclusion of performance bias, which could be associated with the use of human landing catches and further strengthen the quality of evidence deriving from the study. Moreover, the fact that mosquito trapping was conducted during three consecutive days also reduce bias due to variation in rainfall or temperature (Antonio-Nkondjio et al., 2018). Pilot studies conducted so far in Cameroon using aqueous formulation of *Bacillus sphaericus* reported limited impact of the intervention (Hougard et al., 1993; Barbazan et al., 1997). In the present study, the use of VectoMax granules combining both *B. thuringiensis* and *B. sphaericus* which are more stable with a longer residual effect (WHO, 2016) permitted to increase the impact of the intervention.

A significant difference in *Culex* species composition was recorded between larviciding intervention and non-intervention areas. The Shannon index measuring the diversity of species was significantly low in larviciding intervention area when comparing baseline data to data recorded during treatment whereas it was more stable in larviciding non-intervention areasupporting an influence of larviciding on *Culex* species diversity and distribution. Seven *Culex* species were previously recorded in Yaoundé, including *Cx. quinquefasciatus*, *Cx. perfuscus*, *Cx. duttoni*, *Cx. antennatus*, *Cx. poicilipes*, *Cx. univittatus* and *Cx*. (*Lutzia*) *trigripes*, with *Cx. quinquefasciatus* representing over 85% of the total *Culex* mosquitoes (Nchoutpouen et al., 2019). Up to 68 *Culex* species have been reported from Cameroon but the distribution of *Culex* species was found to vary significantly with collection sites or region of the country (Bamou et al., 2021). The poor diversity of species recorded during the present study could be due to the fact that just few species are able to adapt to urban environment (Klinkenberg et al., 2008; Talipouo et al., 2017). *Culex* species are also known to breed in a variety of breeding habitats and to feed on a variety of hosts (humans, mammals, birds) (Talipouo et al., 2017; Nchoutpouen et al., 2019). The low diversity of breeding habitats and hosts in the urban environment could somewhere explain the low diversity of species recorded. Moreover, the fact that non-baited CDC traps were used for mosquito sampling could also explain the low diversity observed during this study, but this deserves further investigations. Significant variation in trap efficiencies was observed between traps placed indoors and outdoors with more *Culex* mosquitoes trapped indoors than outdoors. The following could result from the probable influence of wind on CDC light traps performance or the absence of any bait close to the trap. Similar findings have been recorded in a previous study (Bamou et al., 2018).

The implementation of larviciding activities was associated with over 69% reduction of breeding habitats with *Culex* larvae. This reduction rate is close to the reduction observed for anopheline mosquitoes (Antonio-Nkondjio et al., 2021). Although this was not measured, it is likely that the efficacy of the intervention may have varied according to the type of habitat and ecological factors such as rainfall since significant variation of the efficacy was observed between seasons. The fact that larviciding target mosquito feeding both indoors and outdoors makes this intervention a suitable complement to existing control interventions such as LLINs. This intervention could be particularly efficient in urban settings where most mosquito bites occur outdoors (Doumbe-Belisse et al., 2018; Antonio-Nkondjio et al., 2021).

Although *Culex* mosquitoes in the city of Yaoundé have not been incriminated as vectors of diseases (Nchoutpouen et al., 2019), they always cause important nuisance in urban settings. Moreover, future introduction of arboviruses by *Culex* species in Yaoundé could not be excluded since they are competent vectors of arboviruses such as West Nile or Rift Valley viruses (Bamou et al., 2021).

Therefore, reducing *Culex* densities could improve the acceptability and uptake of malaria vector control interventions by the population and deserve further attention. Local communities usually do not make a difference between anophelines which transmit malaria and other mosquitoes such as *Culex* or *Aedes*.

The present study has two limitations: (i) the fact that mostly standing water collections were targeted and polluted habitats such as pit latrines were not accessible and treated; this might have limited the impact of the intervention on *Culex* mosquitoes; and (ii) the study did not assess the cost-effectiveness of larviciding, which is very important for policymakers.

## 5 Conclusions

This study provided evidence supporting a high impact of larviciding on *Culex* mosquitoes. In the context of increasing epidemiological importance of non-anopheline mosquitoes such as *Culex* and *Aedes*, in addition to the high nuisance they cause, extending malaria control programmes to these mosquitoes could be cost-effective and could improve acceptability and adherence of communities to vector control interventions. In cases where larviciding trials target *Culex* species, the study design should include targeting highly polluted habitats such as pit latrines to improve the impact of the intervention on *Culex* mosquitoes.

## Funding

This work received financial support from 10.13039/100010269Wellcome Trust Senior Fellowship in Public Health and Tropical Medicine (202687/Z/16/Z) to C.A-N. The funding body did not have any role in the design, collection of data, analysis and interpretation, and in writing of the manuscript.

## Ethical approval

This study was conducted according to the guidelines of the Declaration of Helsinki, and approved by the Cameroon National Ethics Committee for research on Human Health, Ethical Clearance N°2016/11/832/CE/CNERSH/SP and by the Ministry of Public Health of Cameroon (Reference: 631-06-17). Also, import permit for the use of VectoMax®G in Cameroon was granted by the Minister of Trade (Reference IF014167; IF021096; IF031126).

## CRediT authorship contribution statement

**Abdou Talipouo:** Methodology, Investigation, Writing – original draft, Writing – review & editing, Software, Formal analysis, Data curation, Visualization. **Patricia Doumbe-Belisse:** Methodology, Investigation, Data curation. **Carmène S. Ngadjeu:** Methodology, Investigation, Data curation. **Landre Djamouko-Djonkam:** Methodology, Investigation, Data curation. **Elysée Nchoutpouen:** Writing – review & editing. **Roland Bamou:** Methodology, Investigation, Data curation. **Nadège Sonhafouo-Chiana:** Methodology, Investigation, Data curation. **Audrey Paul Marie Mayi:** Methodology, Investigation, Data curation, Software, Formal analysis. **Gisèle Aurélie Dadji Foko:** Writing – review & editing. **Parfait Awono-Ambene:** Writing – review & editing, Supervision. **Sévilor Kekeunou:** Writing – review & editing. **Charles S. Wondji:** Writing – review & editing, Supervision, Resources, All authors read and approved the final manuscript. **Christophe Antonio-Nkondjio:** Conceptualization, Methodology, Investigation, Writing – original draft, Supervision, Visualization, Project administration, Funding acquisition.

## Declaration of competing interests

The authors declare that they have no known competing financial interests or personal relationships that could have appeared to influence the work reported in this paper.

## Data Availability

The data supporting the conclusions of this article are included within the article.
